# PDK1-mTOR signaling pathway inhibitors reduce cell proliferation in MK2206 resistant neuroblastoma cells

**DOI:** 10.1186/s12935-015-0239-4

**Published:** 2015-09-29

**Authors:** Lei Qi, Hidemi Toyoda, Dong-qing Xu, Ye Zhou, Naoto Sakurai, Keishirou Amano, Kentaro Kihira, Hiroki Hori, Eiichi Azuma, Yoshihiro Komada

**Affiliations:** Department of Pediatrics and Developmental Science, Mie University Graduate School of Medicine, 2-174 Edobashi, Tsu, Mie 514-8507 Japan; Department of Pediatrics, Xin Hua Hospital Affiliated to Shanghai Jiao Tong University School of Medicine, 1665 Kong Jiang Road, Shanghai, 200092 China; Department of Child Health Nursing, Mie University Graduate School of Medicine, 2-174 Edobashi, Tsu, Mie 514-8507 Japan

**Keywords:** Neuroblastoma, MK2206, Akt, PDK1, mTOR, Cell growth

## Abstract

**Purpose:**

AKT plays a pivotal role in the signal transduction of cancer cells. MK2206, an AKT inhibitor, has been shown to be an effective anti-cancer drug to a variety of cancer cell lines. However, some cancer cells acquire resistance to MK2206 and new strategies to suppress these cell lines remain to be developed.

**Experimental design:**

Acquired MK-2206-resistant neuroblastoma (NB) cell sublines were induced by stepwise escalation of MK-2206 exposure (4–12 weeks). MTT assay was used to validate cell proliferation. Flow cytometry was performed for cell cycle analysis. Western blot assay was used for cell signaling study.

**Results:**

MK2206 (5–10 µmol) significantly suppressed cell growth of MK2206 non-resistant NB cells (LAN-1, KP-N-SIFA, NB-19 and SK-N-DZ), but is less efficient in inhibiting that of resistant sublines, even after 2-week MK2206-free incubation. MK2206 acted in mTOR-S6K dependent and independent methods. MK-2206 resistant sublines (LAN-1-MK, KP-N-SIFA-MK, and SK-N-DZ-MK) showed lower IC_50_ of GSK2334470 (PDK1 inhibitor). The cell growth of all sublines was prohibited by AZD8805 (mTOR inhibitor), with IC_50_ of AZD8805 3–10 times lower than MK2206 non-resistant cells. The signaling profiles of these resistant sublines were characterized by elevated PDK1-mTOR-S6K activity, accompanying by low phosphorylation of AKT compared with non-resistant counterparts. GSK2334470 and AZD8055 effectively inhibited phosphorylation of PDK1 and mTOR, respectively, and induced higher G0–G1 ratio in LAN-1-MK than that in LAN-1 as well. PDK1 and mTOR inhibitors effected on phosphorylation of GSK3β in some of resistant sublines.

**Conclusion:**

NB cells can acquire MK2206 resistance after exposure for 4–12 weeks. Resistant cells feature reliance on PDK1-mTOR-S6K pathway and are more sensitive to PDK1 and mTOR inhibitors than the non-resistant counterparts. Thus, suppression of PDK1-mTOR-S6K signaling pathway is an effective way to overcome the MK2206 resistance, and this may be a promising strategy for targeted therapy.

## Background

The mechanisms underlying cancer are marked by complex aberrations that activate critical cellular signaling pathways in tumorigenesis. The phosphatidylinositol 3-kinase/protein kinase-B/mammalian target of rapamycin (PI3K/AKT/mTOR) signaling cascade is one of the most important intracellular pathways, which is frequently activated in diverse cancers [1–3]. Activation of the PI3K/AKT/mTOR signaling pathway mediated through molecular aberrations is instrumental in promoting tumor development as well as resistance to anticancer therapies [4]. The PI3K/AKT/mTOR pathway can be activated by transmembrane tyrosine kinase growth factor receptors, such as ErbB family receptors, fibroblast growth factor receptors (FGFR), insulin-like growth factor 1 receptor (IGF-1R), and others [5, 6]. In addition, G protein-coupled receptors such as activated RAS can stimulate PI3K through its catalytic subunit [7]. Mutations in PI3K/AKT/mTOR signaling can associate with disorders with a high incidence of cancers [8]. These mechanisms leading to aberrant PI3K/AKT/mTOR signaling in affected cancer cells become the molecular targets of cancer therapy [9, 10].

Numerous efforts have been made to develop PI3K/AKT/mTOR targeted therapies for cancer treatments. Various drugs such as PI3K, AKT, or mTOR kinase inhibitors are in clinical development, and have been approved by the Food and Drug Administration (FDA) and the European Medicines Agency (EMA) for treating cancers, such as advanced renal cell cancer [11, 12], hormone receptor-positive [13], HER2-negative breast cancer, and neuroendocrine tumors of pancreatic origin [14]. AKT/PKB (protein kinase-B) is a family composed of three serine/threonine kinases known as AKT1, AKT2 and AKT3. AKT is an important part of PI3K signaling as the activation of the protein is caused by PI3K and PDK1 mediated phosphorylation in the catalytic domain at threonine 308. AKT activation is involved in tumor progression through increased cell proliferation and survival, invasion, metabolism or angiogenesis [15]. AKT regulates downstream targets in the PI3K pathway such as TSC2 (which leads to activation of mTORC1) and outside of the PI3K pathway such as Bcl-2-associated proteins, glycogen synthase kinase-3β (GSK3β) or mouse double minute 2 homolog (MDM2). Inhibition of AKT as an important node in PI3K signaling could have a significant impact on tumors that are addicted to PI3K/AKT/mTOR axis activity [1].

MK-2206 is an investigational allosteric inhibitor of AKT that requires the PH domain of AKT for activity, but does not interact with the ATP binding pocket. As a result, MK-2206 is highly selective for AKT inhibition, has higher potency against recombinant human AKT1 and AKT2 isoforms than AKT3, has little off-target kinase activities, and is less vulnerable to feedback activation of AKT compared with ATP-competitive inhibitors [16]. As a single agent, and in combination with cytotoxic drug, MK-2206 is being tested to be effective both in vitro and in vivo; both in adult tumors [17–22] and in spectrum of pediatric tumors [23, 24]. In clinical trial, stable disease was observed in different cancers [25, 26]. Phase I Studies showed doses of MK-2206 ranging between 0.25 and 100 mg oral were well tolerated [27]. A larger phase I exploration is underway, examining MK-2206 dose formulations and combinations with cytotoxic drug [24, 28].

PDK1 (3-phosphoinositide-dependent protein kinase 1) activates a group of protein kinases belonging to the AGC [PKA (protein kinase A)/PKG (protein kinase G)/PKC (protein kinase C)]-kinase family that play important roles in mediating diverse biological processes [29, 30]. Many cancer-driving mutations induce activation of PDK1 targets including AKT [31], S6K (p70 ribosomal S6Kinase) [32] and SGK (serum- and glucocorticoid-induced protein kinase) [33]. Small molecule GSK2334470 inhibits PDK1 with an IC_50_ of ~10 nM, but does not suppress the activity of 93 other protein kinases including 13 AGC-kinases most related to PDK1 at 500-fold higher concentrations [34]. Addition of GSK2334470 ablated T-loop residue phosphorylation and activation of SGK isoforms and S6K1 induced by serum or IGF-1 (insulin-like growth factor 1) [35].

Mammalian target of rapamycin (mTOR) is also a serine/threonine protein kinase, modulating cancer cell proliferation, mortality, survival and protein synthesis [36]. AZD8055 is a novel and potent ATP-competitive mTOR inhibitor, which blocks both mTORC1 and mTORC2 activation [37], effecting on cancer cell growth and survival [38–40]. AZD8055 is a first-in-class orally available, potent and specific inhibitor of mTOR kinase activity, and shows a promise for suppressing tumor growth [41].

Establishment of resistant sublines is a common experimental method to investigate the mechanism of drug resistance. AKT inhibitor, such as MK-2206, shows promising effect on cancer therapy, but MK-2206 resistance remains to be studied. In this study, we successfully induced MK-2206-resistant sublines by using stepwise exposure, and identified cell signaling features of resistant sublines. The purpose of our study is to investigate the acquired mechanism of resistance to AKT inhibition and to explore strategies to overcome the tolerance of the AKT inhibitors in cancer therapy.

## Methods

### Cell lines and cell culture

The following four human NB cell lines were used and evaluated in this study. They are NB-19 [42], KP-N-SIFA [43], SK-N-DZ [44] and LAN-1 [45], which were studied in our previous papers [46, 47]. All these 4 cell lines were cultured in RPMI1640 (R8758, Sigma) medium supplemented with 10 % fetal bovine serum (FBS) (GIBCO). Cells were incubated in a humidified atmosphere at 37 °C with 5 % CO_2_.

### Induction of MK-2206-resistant NB cells

To induce acquired MK-2206-resistance, aliquots of parent cells were seeded into 25 cm^2^ culture bottles, and cultured in 10 % FBS RPMI1640 medium with stepwise escalation of concentration of MK-2206 (from 1 to 5 μg). Fresh medium with MK-2206 was changed every 72 h. Cells were transferred into new culture bottles every 7 days. We continued this process while observing cell death every day, and performing cell counting by using Invitrogen Cell Counter regularly. Escalation of MK-2206 was performed after twice solid cell counting results showing increasing cell number. Thus, after 4–12 weeks, MK-2206-resistant sublines were obtained that grew stably in MK-2206 (5 μg)-containing medium, and these resistant cells were named LAN-1-MK, NB-19-MK, KP-N-SIFA-MK, and SK-N-DZ-MK.

### Antibodies and reagents

The following antibodies and reagents were used in the present study: anti-AKT (#9272, Cell Signaling), anti-phospho-AKT (Ser473) (#4058, Cell Signaling), phospho-PDK1 (Ser241) (C49H2) (#3438, Cell Signaling), phospho-mTOR (Ser2448) (#5536, Cell Signaling), phospho-p70 S6Kinase (Thr389) (#9205, Cell Signaling), anti-GSK-3β (27C10) (#9315, Cell Signaling), p-Ser9-GSK-3-beta (#9322, Cell Signaling), anti-N-MYC(C-19) (SC-791, Santa Cruz Biotechnology), and anti- c-Myc (9E10) (sc-40, Santa Cruz Biotechnology).

MK-2206 (AKT inhibitor, A10003) was purchased from Selleckchem. GSK2334470 (PDK1 inhibitor, No. 4143) was purchased from Tocris Bioscience. AZD8055 (mTOR inhibitor, 1009298-09-2) was purchased from LC Laboratories. U0126 (MEK inhibitor, 70970-5) was purchased from Cayman Chemical.

### Cell counting

MTT cell counting reagent was obtained from Sigma-Aldrich. Cells (5 × 10^3^) were seeded in 100 μl medium in 96 wells plates and pre-incubated for 6 h before the addition of inhibitors. MTT (20 μl, 5 mg/ml) was added into each well. After 3.5 h incubation in a humidified atmosphere at 37 °C with 5 % CO_2_, carefully removed media, and added DMSO (150 μl), then shaking for 15 min. The absorbance at 590 nm was measured using multi-spectrophotometer (Viento, Dainippon, Japan). The optical density was then used to extrapolate in the cell number from a standard curve. The standard curves were drawn for each cell line for each type of medium. The results are expressed as mean ± SD from 3 independent experiments.

### Western blotting

Cytoplasmic extracts were obtained as previously reported [48]. The proteins (40 μg/lane) were run on 7.5, 10 or 15 % sodium dodecyl sulfate–polyacrylamide gel electrophoresis followed by semi-dry transfer to PVDF membrane (Invitrogen, Carlsbad, CA). Transferred PVDF blots were pretreated with 5 % non-fat dry milk in TBST containing 0.1 % Tween-20 and incubated with primary antibody (1:1000–3000) at 4 °C overnight. The membrane was then washed 3 times with TBST and incubated with horseradish peroxidase-conjugated secondary antibody (1:1000–3000) for 1 h at room temperature. For phosphorylated protein, transferred PVDF blots were pretreated with PVDF Blocking Reagent (TOYOBO, Osaka, Japan) for 1 h, and incubated with primary and then with secondary antibody (1: 3000–6000) which were diluted with Can Get Signal^®^ Immunoreaction Enhancer Solution (TOYOBO, Osaka, Japan) at room temperature for 1 h. After washing three times again, antibodies bound to protein blots were detected by using Western Lightening Chemiluminescence Reagent Plus (Perkin Elmer Life Science, Boston, MA, USA), visualized on LAS-3000 mini (FUJIFILM). The blots were quantitated and cropped using Multi Gauge Ver 3.0 (FUJIFILM).

### Cell cycle analysis

Cell cycle analysis was performed after treatment with/without MK-2206 for 12 h. Cells (2 × 10^6^) were harvested and fixed in 99.5 % ethanol over night at −20 °C, followed by incubation with 500 μl propidium iodide (PI) Triton X-100 solution containing RNase A at room temperature for 30 min in darkness, then the DNA content was analyzed immediately with FACScan flow cytometer,analyzed by using ModFitLT software.

### Statistical analyses

A two-sided paired *t* test was used to determine statistical significance. A P < 0.05 was considered as statistically significant.

## Result

### MK-2206 sensitivity and acquired MK-2206 resistance in NB cell lines

**Fig. 1:**
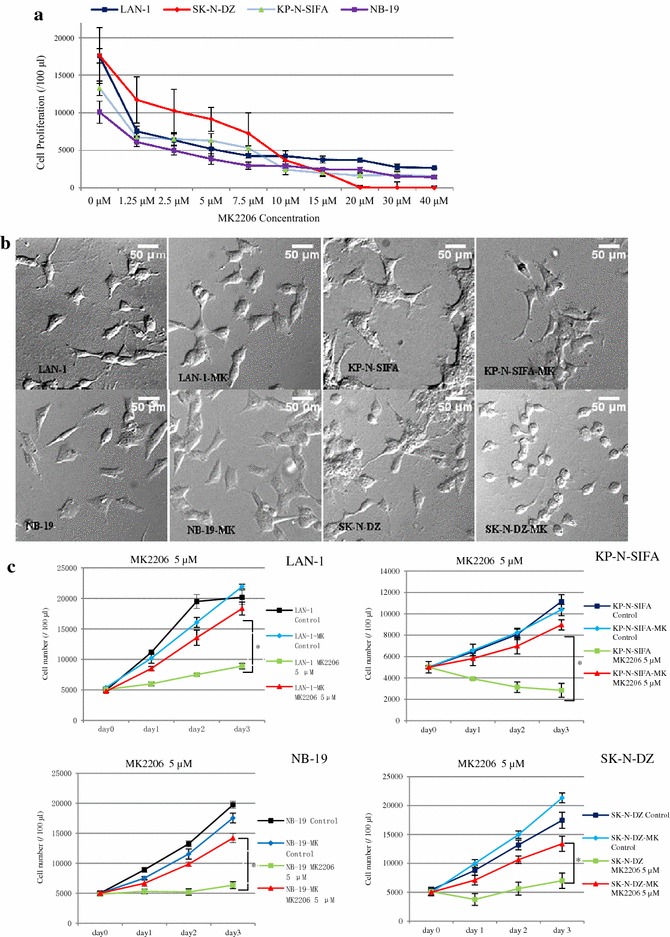
MK-2206 suppressed the cell growth of NB cells. **a** MK-2206 suppressed the cell growth of NB cell lines. LAN-1, KP-N-SIFA, NB-19, and SK-N-DZ cells were cultured in RPMI1640 + 10 % FBS with MK-2206 at indicated concentrations. Cell growth was evaluated as cell numbers at 72 h, and it was repeated three times. Data are expressed as the mean (±SD). **b** Photomicrographs of MK-2206 non-resistant and resistant cells. Cells were cultured in glass bottom slide chambers with RPMI1640 + 10 % FBS, with MK-2206 (resistant sublines)/without MK-2206 (non-resistant cells) overnight. A 50 µm scale is indicated (Olympus Fluoview fv1000, DIC acquisition, ×40)

**Fig. 2:**
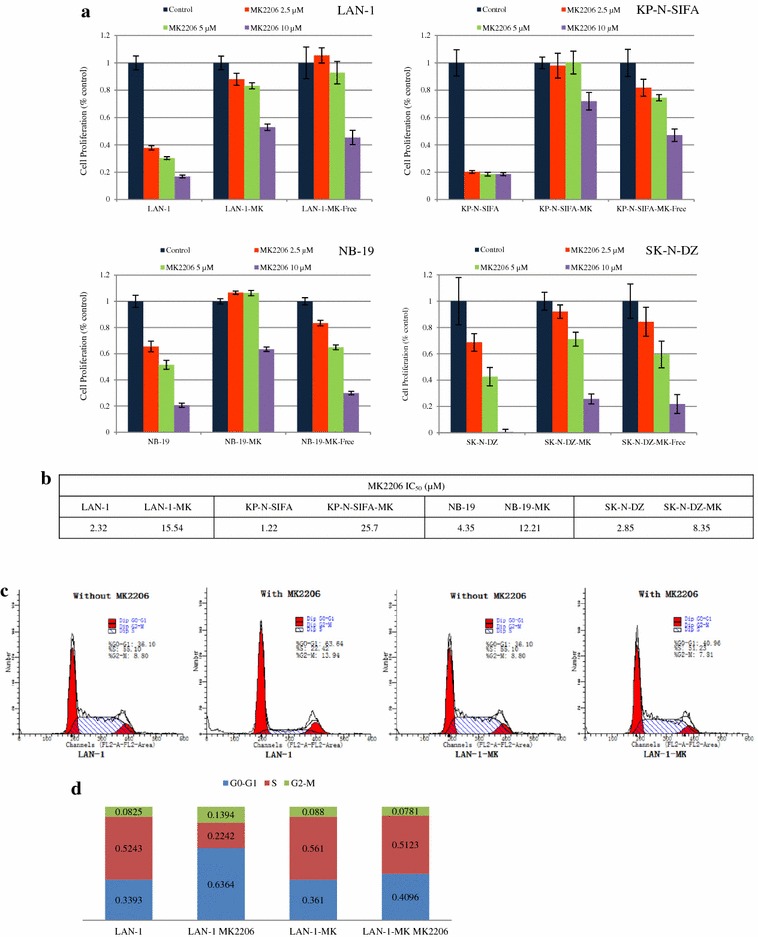
MK-2206 showed less inhibition in cell growth of MK-2206-resistant sublines. **a** MK-2206 showed less inhibition in the proliferation of MK-2206-resistant sublines than in the non-resistant cells. Indicated cells were cultured in RPMI1640 + 10 % FBS with MK-2206 at indicated concentrations. Cell growth was evaluated as cell numbers at indicated hours, and it was repeated three times. Data are expressed as the mean (±SD). *P < 0.01. **b** MK2206 suppressed cell growth in a dose dependent method, and MK-2206-resistant sublines maintained resistance after 2-week withdrawal of MK-2206. Indicated cells were cultured in RPMI1640 + 10 % FBS with MK-2206 at the indicated concentrations. Cell growth was evaluated as cell numbers at 72 h, and it was repeated three times. Data are expressed as the mean (±SD)

To study the inhibitory effect of MK-2206 on NB cell growth, cells (LAN-1, NB-19, KP-N-SIFA, and SK-N-DZ) were selected and treated with MK-2206 at indicated concentrations for 72 h. MK-2206 treatment induced a dose dependent inhibition of cell proliferation, with IC_50_ ranging from 1.22 μM (KP-N-SIFA) to 4.35 μM (NB-19) (Figs. 1a and 2b). These cells were deined as MK-2206 non-resistant cells.

**Fig. 3:**
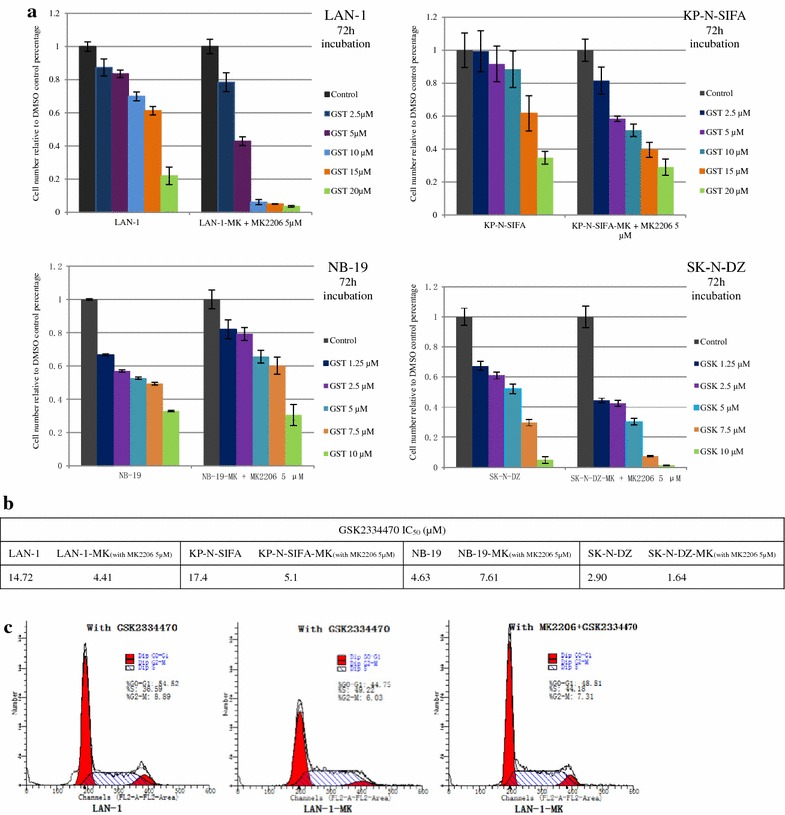
Effect of GSK2334470 (GSK) on PDK1-mTOR-S6K axis in MK-2206-resistant sublines. **a**–**d** After 1 h serum starvation, indicated cells were incubated in RPMI1640 + 10 % FBS with/without MK-2206 (5 μM) or GSK (5 μM). Phosphorylation of PDK1, AKT, mTOR, and S6K were detected by western blot at 1.5 and 12 h, so were AKT and Actin. GSK3β, p-GSK3β and N-MYC were also detected

To explore acquired MK-2206 resistance in NB cells, stepwise escalation of MK-2206 exposure (4–12 weeks) was applied to induce MK-2206 resistance. MK-2206-resistant sublines (LAN-1-MK, NB-19-MK, KP-N-SIFA-MK, and SK-N-DZ-MK) proliferated during 72 h incubation in RPMI1640 plus 10 % FBS medium in the presence of MK-2206 (5 μM), when non-resistant cell number declined, and difference were significant (Fig. 1c). MK-2206 suppressed cell growth in a dose dependent method, and significant difference was observed between MK-2206 non-resistant cells and resistant sublines in RPMI1640 plus 10 % FBS medium with MK-2206 at each indicated concentrations (Fig. 2a). MK-2206 IC_50_ of resistant sublines ranged from 8.35 μM (SK-N-DZ-MK) to 25.7 μM (KP-N-SIFA-MK) (Fig. 2b). Furthermore, 2 weeks of MK-2206-free culture could not completely restore the sensitivity of MK-2206 in the resistant sublines, named as LAN-1-MK-Free, NB-19-MK-Free, KP-N-SIFA-MK-Free, and SK-N-DZ-MK-Free (Fig. 2a).

Additionally, we compared morphologies of MK-2206 non-resistant and resistant cells. LAN-1 and SK-N-DZ were reported to be N type cells [49, 50]. In our study, MK-2206 non-resistant cells and resistant sublines showed a very similar phenotype in culture, characterized by variable shape, short neurite processes formation, and with no apparent directional orientation. Only exception is SK-N-DZ-MK, which showed smaller and rounder comparing with its MK-2206 non-resistant opponent cell (Fig. 1b).

MK-2206 was reported to affect cell-cycle distribution [51]. In our study, cell-cycle analysis showed that MK-2206 (5 μM) caused G0–G1 accumulation from 33.93 to 63.64 % in LAN-1 cells, but not in LAN-1-MK subline (Fig. 2c, d).

### PDK1 inhibitor (GSK2334470) prohibited cell growth of MK-2206-resistant sublines

PDK1, regulated by PI3K, phosphorylates and activates the AGC kinase members (protein kinase A, G, and C), including AKT and mTOR [30]. In this study, to assess PDK1 activity in MK-2206 resistance, non-resistant cells and resistant sublines were treated with/without GSK2334470 (PDK1 inhibitor) for 72 h, and cell viability was detected by MTT assay. GSK2334470 attenuated cell growth of non-resistant cells (LAN-1, KP-N-SIFA, NB-19, and SK-N-DZ), and more of resistant sublines (LAN-1-MK, KP-N-SIFA-MK, and SK-N-DZ-MK), combined with MK-2206 (5 μM), in a dose dependent method, which were significantly different at each indicated concentration (Fig. 3a). IC_50_ of GSK2334470 in MK-2206-resistant sublines ranged from 1.64 (SK-N-DZ-MK) to 5.1 μM (KP-N-SIFA-MK), which was lower than that of MK-2206 non-resistant cells with the IC_50_ ranging from 2.90 (SK-N-DZ) to 17.4 μM (KP-N-SIFA) (Fig. 3b).

**Fig. 4:**
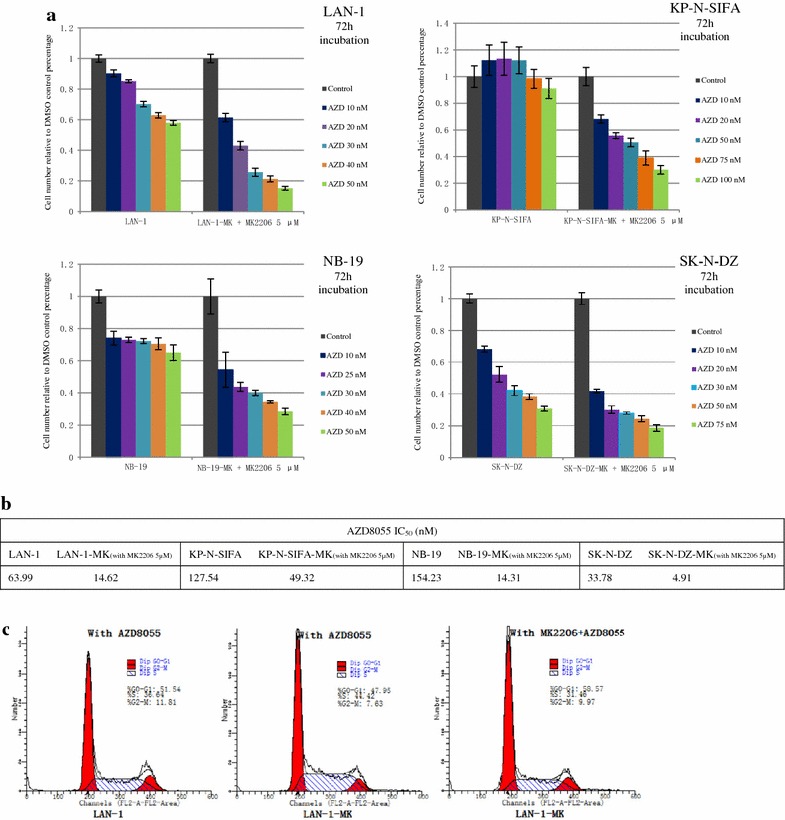
Effect of GSK2334470 (GSK), PDK1 inhibitor, in MK-2206-resistant sublines compared with non-resistant cells. **a** Indicated cells were treated with GSK at indicated concentrations, with/without MK-2206 (5 μM) in RPMI1640 + 10 % FBS. Cell growth was evaluated as cell numbers at 72 h, and it was repeated three times. Data are expressed as the mean (±SD). **b** The effect of GSK on cell cycle phase distribution in LAN-1 and LAN-MK. LAN-1 and LAN-1-MK were treated with GSK (5 μM) with/without MK-2206 (5 μM) in RPMI1640 with 10 % FBS for 12 h followed by analysis of cell cycle phase distribution, as introduced in methods and material. Indicated cells were stained with PI for 30 min followed by FACScan flow cytometer. **c** IC_50_ of GSK in indicated cells

GSK2334470 induced G0–G1 accumulation of cell cycle phase distribution in LAN-1 increasing from 39.33 to 54.52 % (Figs. 2c, 3c). In LAN-1-MK, GSK2334470 induced G0–G1 accumulation increasing from 36.10 to 44.75 % without MK-2206, and from 40.91 to 48.51 % with MK-2206 (Figs. 2b, 3c).

### The mTOR inhibitor (AZD8055) prohibited cell growth of MK-2206-resistant sublines

Signaling pathway of PI3K/AKT/mTOR is frequently dysregulated in different disorders of cell growth, survival, and resistance. To study the role of mTOR in the MK-2206 resistance, MK-2206 non-resistant cells and resistant sublines were treated with AZD8055 (mTOR inhibitor) for 72 h, and cell viability was detected by MTT assay. AZD8055 suppressed cell growth of non-resistant cells (LAN-1, KP-N-SIFA, NB-19, and SK-N-DZ), and more of resistant sublines (LAN-1-MK, KP-N-SIFA-MK, NB-19-MK and SK-N-DZ-MK) in a dose dependent method, combined with MK-2206 (5 μM), which were significantly different at each indicated concentration (Fig. 4a). AZD8055 prohibited cell growth of LAN-1, NB-19, KP-N-SIFA, and SK-N-DZ with IC_50_ ranging from 33.78 (SK-N-DZ) to 154.23 nM (NB-19), while IC_50_ of AZD8055 of MK-2206 resistant sublines ranged from 4.91 nM (SK-N-DZ-MK) to 49.32 nM (KP-N-SIFA-MK) (Fig. 4b).

**Fig. 5:**
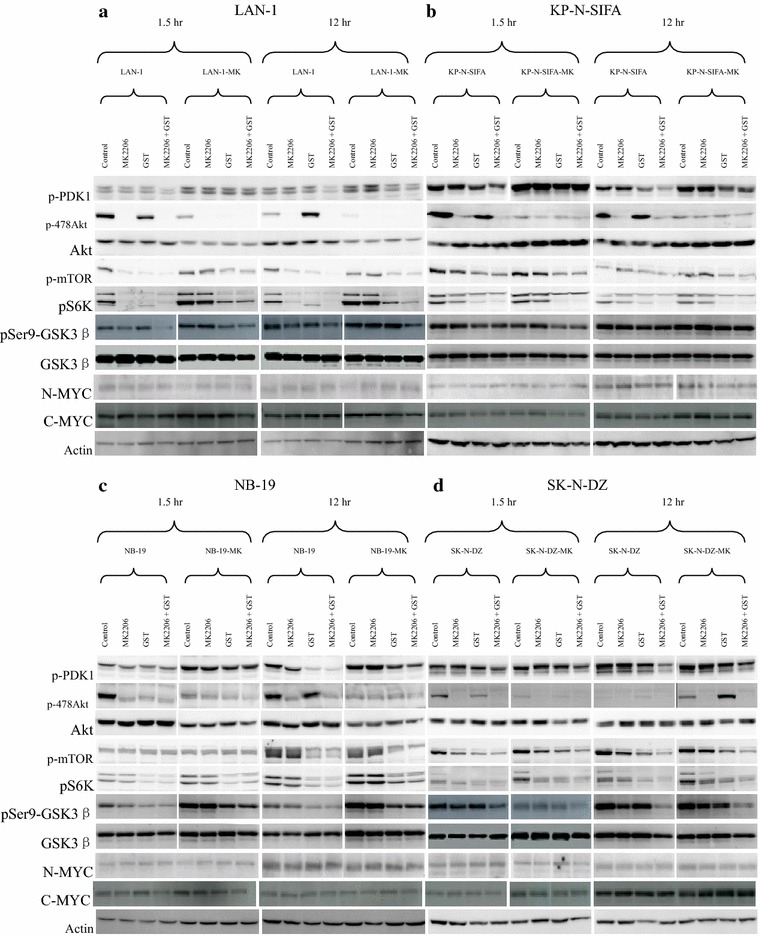
Effect of AZD8055 (AZD), mTOR inhibitor, in MK-2206-resistant sublines compared with non-resistant cells. **a** Indicated cell lines were treated with AZD at indicated concentrations, with/without MK-2206 (5 μM) in RPMI1640 + 10 % FBS. Cell growth was evaluated as cell numbers at 72 h, and it was repeated three times. Data are expressed as the mean (±SD). **b** The effect of AZD on cell cycle phase distribution in LAN-1 and LAN-MK. Indicated cells were treated with AZD (50 nM) with/without MK-2206 (5 μM) in RPMI1640 with 10 % FBS for 12 h followed by analysis of cell cycle phase distribution, as introduced in methods and material. Cells were stained with PI for 30 min followed by FACScan flow cytometer. **c** IC_50_ of AZD of indicated cell lines

**Fig. 6:**
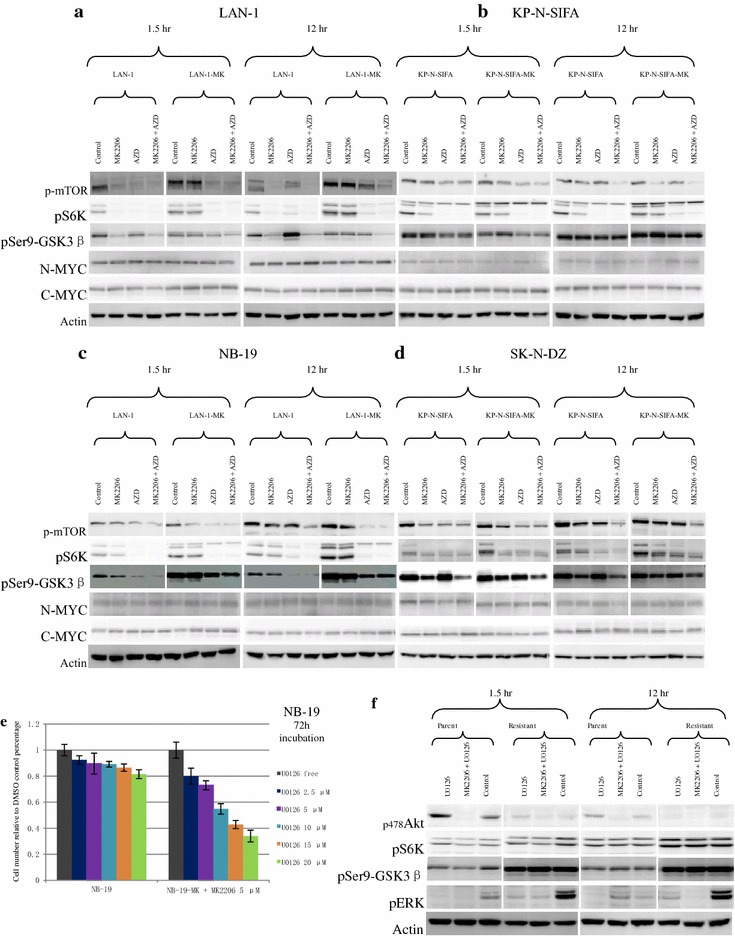
Effect of AZD8055 (AZD) on mTOR-S6K axis in MK-2206-resistant sublines. **a**–**d** After 1 h serum starvation, indicated cell lines were incubated in RPMI1640 + 10 % FBS with/without MK-2206 (5 μM) or AZD (50 nM). Phosphorylation of mTOR and S6K were detected by western blot at 1.5 and 12 h, so was Actin. **e** Effect of U0126 on NB-19-MK cell line compared with NB-19. Indicated cells were treated with U0126 at indicated concentrations in RPMI1640 + 10 % FBS, with/without MK-2206 (5 μM). Cell growth was evaluated as cell number at 72 h, and result was repeated three times. Data are expressed as the mean (±SD). **f** NB-19 and NB-19-MK were incubated in RPMI1640 + 10 % FBS with/without MK-2206 (5 μM) or U0126 (5 μM). Phosphorylation of ERK, AKT, and S6K were detected by western blot at 1.5 and 12 h, so was Actin

AZD8055 induced G0–G1 accumulation in LAN-1cell cycle phase distribution from 39.33 to 51.54 % (Figs. 2c, 4c). AZD8055 also induced an increase in G0–G1 phase from 36.10 to 47.95 % without MK-2206 and from 40.91 to 58.57 % with MK-2206 in LAN-1-MK (Figs. 2c, 4c).

### Decrease in AKT phosphorylation in MK-2206-resistant sublines

To study inhibitory effect of MK-2206 on AKT pathway, cells were culture in RPMI + 10 % FBS with/without MK-2206, after 1 h serum starvation. P-AKT was detected by an anti-p-AKT antibody. AKT phosphorylation was suppressed by 5 μM of MK-2206 in LAN-1, KP-N-SIFA, NB-19, and SK-N-DZ cells (Fig. 5a–d). Stepwise escalation of MK-2206 exposure reduced p-AKT and total AKT levels in LAN-1-MK, NB-19-MK and SK-N-DZ-MK, comparing with the non-resistant opponents, after 1.5 and 12 h incubation without MK-2206 (Fig. 5a, c, d). KP-N-SIFA-MK had low levels of p-AKT but high levels of total AKT than its non-resistant counterpart (Fig. 5b).

### AKT acted in mTOR-S6K dependent and independent manners in NB cells 

In LAN-1 and SK-N-DZ, phosphorylation of mTOR and S6K (p-mTOR and p-S6K) were reduced by MK-2206 at 5 μM; in LAN-1-MK and SK-N-DZ-MK, p-mTOR and p-S6K were higher than those in their non-resistant counterparts, and not suppressed by MK-2206 at 5 μM (Fig. 5a, d). In the meantime, p-mTOR and p-S6K were not affected by MK-2206 (5 μM) in NB-19, KP-N-SIFA and their resistant sublines (Fig. 5b, c). It suggested that AKT acted in mTOR-S6K dependent method (LAN-1 and SK-N-DZ), in which inhibition of AKT reduced activity of mTOR-S6K signaling; mTOR-S6K independent method (KP-N-SIFA and NB-19), in which inhibition of AKT did not impair the phosphorylation of mTOR-S6K signaling.

### PDK1 inhibitor (GSK2334470) suppressed mTOR-S6K pathway in MK-2206-resistant sublines

To study the inhibitory effect of GSK2334470 on mTOR-S6K pathway, non-resistant cells (LAN-1, NB-19, KP-N-SIFA, and SK-N-DZ) and the resistant sublines (LAN-1-MK, NB-19-MK, KP-N-SIFA-MK, and SK-N-DZ-MK) were treated with GSK2334470 at 5 μM for 1.5 and 12 h in 10 % FBS medium with/without MK-2206 (5 μM). Decrease of phosphorylated PDK1, mTOR, and S6K (p-PDK1, p-mTOR, and p-S6K) was detected in both MK-2206 non-resistant cells and resistant sublines after 12 hours treatment of GSK2334470 (5 μM) (Fig. 5a–d). MK-2206-resistant sublines had relative high levels of p-PDK1 than the non-resistant sublines (Fig. 5a–d). GSK2334470 suppressed p-mTOR and p-S6K in both MK-2206 non-resistant cells (LAN-1, KP-N-SIFA, NB-19 and SK-N-DZ) and their resistant sublines (Fig. 5a–d). It indicated that PDK1 played an important role in mTOR-S6K signaling with or without AKT activity.

### MTOR inhibitor (AZD8055) suppressed mTOR-S6K pathway in MK-2206-resistant sublines

To study the inhibition effect of AZD8055 on mTOR-S6K pathway, MK-2206 non-resistant cells and resistant sublines were cultured with/without AZD8055 at 50 nM in 10 % FBS medium with/without MK-2206 for 1.5 and 12 h, and phosphorylation of mTOR-S6K signaling was tested by western blot. A decrease in p-mTOR was detected in MK-2206 non-resistant cells and resistant sublines with AZD8055. P-S6K was also inhibited by AZD8055 in these cell lines, including NB-19-MK (Fig. 6a–d).

### GSK-3β and N-MYC expressions in MK-2206-resistant cells

GSK3β (glycogen synthase kinase 3β) is expressed ubiquitously and highly conserved, whose activities is regulated by diverse stimuli and signaling pathways. GSK3β (S9) is mainly phosphorylated and inactivated by PI3K-AKT signal pathway; Raf/MEK and other pathways may also induce the phosphorylation and inactivity of GSK3β [52]. To see if GSK3β is a potential component in MK-2206 resistance, we tested GSK3β and pS9-GSK3β in both MK-2206 non-resistant cells and resistant sublines. The result showed that phosphorylation of GSK3β has less relationship with AKT activity in KP-N-SIFA and SK-N-DZ, because MK-2206 barely reduced pS9-GSK3β (Fig. 4b, d). In LAN-1-MK and NB-19-MK, pS9-GSK3β level did not show any change with/without MK-2206, but their MK-2206 non-resistant opponents did show decline with MK-2206 co-incubation (Fig. 5a, c). Nevertheless, pS9-GSK3β of LAN-1-MK and NB-19-MK was suppressed by PDK1 and mTOR inhibitors (Figs. 5a, c, 6a, c), indicating compensation of PDK1 or mTOR in absence of AKT phosphorylase activity.

Transcription factors of N-MYC and C-MYC were previously shown to contribute to tumourigenesis and progression in NB [53, 54]. LAN-1, NB-19, and SK-N-DZ were reported high N-MYC expression [50]. MYC activity is usually regulated by GSK3β. In our study, expressions of N-MYC and C-MYC did not show solid relationship between pS9-GSK3β and MK-2206 resistance (Figs. 5, 6).

### MAPK inhibitor (U0126) prohibited cell growth in NB-19-MK cells

As cell growth of NB-19-MK was not sensitive to GSK2334470, it is possible that other signal pathway might be involved in MK-2206 resistance. MAPK inhibitor (U0126) effectively suppressed cell growth of NB-19-MK, which U0126 at 5 μM significantly suppressed cell growth of NB-19-MK compared with NB-19 (Fig. 6e), but it did not affect p-S6K (Fig. 6f), indicating MAPK related resistance.

## Discussion

**Fig. 7:**
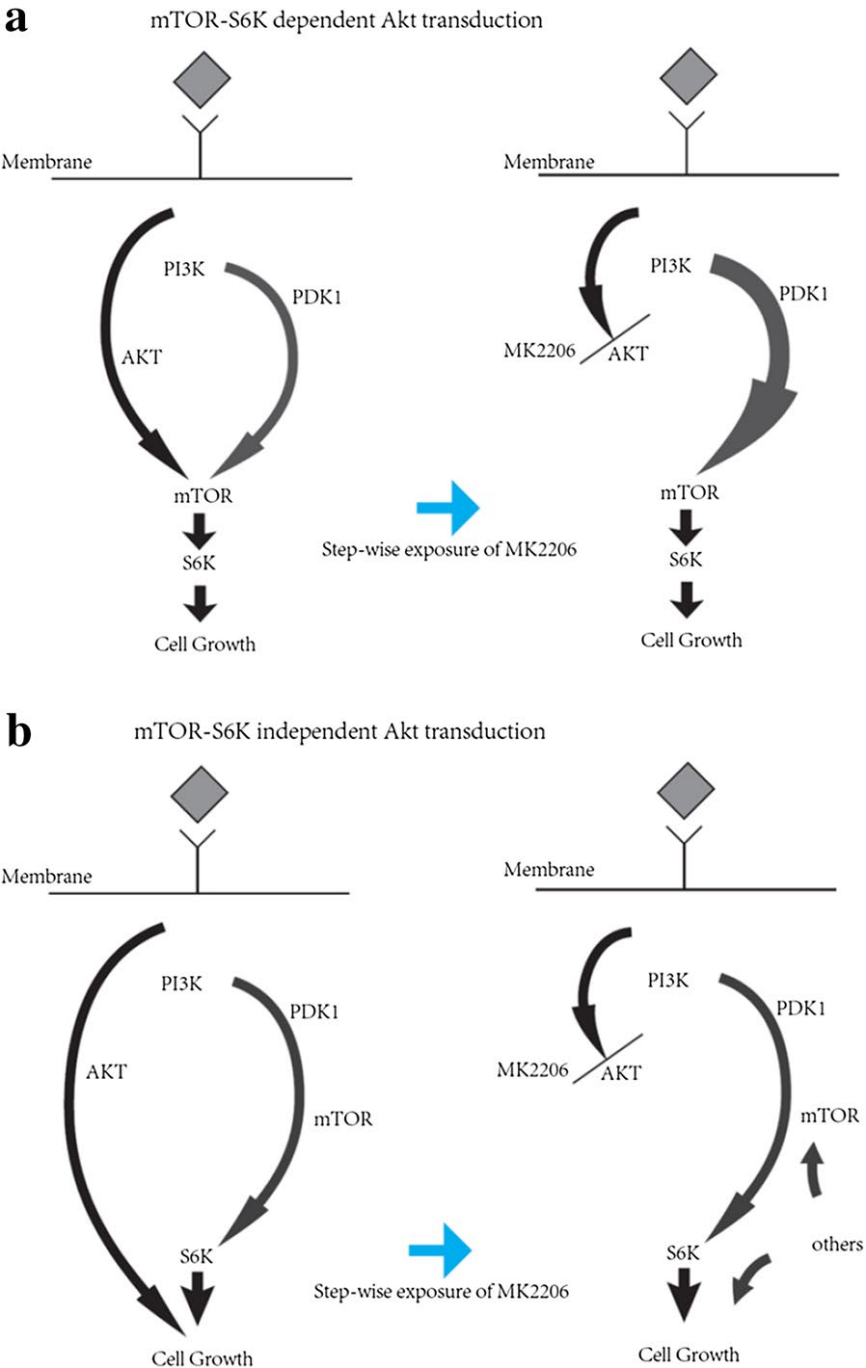
Schematic pathway alternations of MK-2206 resistance. **a** Signal pathway study of MK-2206 non-resistant cells showed that AKT acted in mTOR-S6K dependent method (LAN-1 and SK-N-DZ), in which inhibition of AKT reduced activity of mTOR-S6K signaling. Activity of PDK1 took the place of AKT, and elevated PDK1-mTOR-S6K transduction contributed to MK-2206 resistance, when AKT-mTOR-S6K was blocked. **b** AKT acted in mTOR-S6K independent method (KP-N-SIFA and NB-19), in which inhibition of AKT did not impair the activity of mTOR-S6K signaling. Parallel signaling pathway of PDK1-MTOR-S6K became an important alternative for cell growth of MK-2206-resistant sublines, when AKT was inhibited. And some other signal pathway may contribute to MK-2206 resistance as well

In this study, MK-2206 showed inhibitory effect on cell growth of NB cell lines. Signal pathway study of MK-2206 non-resistant cells showed that AKT acted in mTOR-S6K dependent method (LAN-1 and SK-N-DZ), in which inhibition of AKT reduced activity of mTOR-S6K signaling; and mTOR-S6K independent method (KP-N-SIFA and NB-19), in which inhibition of AKT did not impair the activity of mTOR-S6K signaling. Step-wise exposure to MK-2206 induced acquired resistance in non-resistant cells. MK-2206-resistant sublines showed much lower IC_50_ of PDK1 and mTOR inhibitors, especially mTOR inhibitor (AZD8055). Signal pathway study of resistant sublines showed that LAN-1-MK and SK-N-DZ-MK expressed elevated PDK1-mTOR-S6K transduction (Fig. 7a), which indicated that activity of PDK1 took the place of AKT, and PDK1-mTOR-S6K signaling made contribution to cell growth of their resistant sublines. KP-N-SIFA-MK did not show amplification of mTOR-S6K signaling, but its elevated sensitivity to PDK1 and mTOR inhibitors implied that PDK1-mTOR-S6K transduction became an important alternative for cell growth of KP-N-SIFA-MK. Cell growth of NB-19-MK was related to MAPK pathway (Fig. 7b). Thus, continuous administration inducing signaling pathway alternation may offset MK-2206 inhibitory effect on cell growth, and MK-2206 resistance was not be overcome by short period interruption of administration (2 weeks). These observations suggest that NB cells may be able to enact mechanism base on signal transduction alternation to NB cell survive and escape cell death from singly MK-2206 exposure.

AKT acts as an important role in cell signal transduction, accelerating cancer cell growth. MK-2206 is an allosteric inhibitor of AKT, proved to be an effective reagent to suppress cancer cell growth [22, 25, 26], with synergy observed in combination other targeted therapies in preclinical models [18, 26]. According to our observation, singly exposure MK-2206 may induce acquired resistance of NB cells in 4–12 weeks. Combination with PDK1 or mTOR inhibitor is feasible method to compensate MK-2206 resistance in NB cells. Since in most tumors multiple signaling pathways are involved, many of the inhibitors in clinical development are designed to affect a wide range of targeted kinases [55]. Multi-targeted tyrosine kinase inhibitors are promising to resolve similar resistance. It was reported that the strategy of dual PI3K and mTOR inhibition targets the pathway at two different levels with the potential to effectively overcome the feedback inhibition ordinary observed when mTORC1 inhibitors are used alone, which limits their efficacy [56].

PDK1 is considered as the master upstream regulator of AGC kinase signal transduction, which phosphorylates and activates a diverse set of AGC kinase members regulated by PI3-kinase, when the interaction of insulin and growth factors with their receptors on the outside surface of a cell [30]. PDK1 is constitutively active, thus, AKT and S6K can be activated downstream of PI3K by PDK1. GSK2334470 not only inhibits T-loop phosphorylation and activation of AKT but also those of the RSK2 (p90 ribosomal S6Kinase2) [35]. Our observation showed that PDK1 inhibitor (GSK2334470) is effective in suppressing cell growth of both MK-2206-resistant and non-resistant cells. Suppression of PDK1 affected mTOR-S6K signaling in all MK-2206 non-resistant cells and resistant sublines. In the meantime, GSK2334470 not only prohibited cell growth but induced G0–G1 cell cycle accumulation as the effect of MK-2206 in non-resistant cells. These indicated that PDK1 could be important target in NB therapy.

In our observation, alternation of signaling pathways is the main reason of MK-2206 resistance, including PDK1-mTOR-S6K pathway activation (LAN-1-MK, SK-N-DZ-MK, and KP-N-SIFA-MK), MAPK pathway activation (NB-19-MK). Interestingly, mTOR-S6K works as the mutual signal transduction in MK-2206-resistant sublines, because mTOR inhibitor, AZD8055, is effective to cell growth of these MK-2206-resistant sublines. Dysregulation of mTOR signaling occurs in various tumor types, including breast cancer, and has been associated with cancer pathogenesis, disease progression, and treatment resistance [57]. Signaling transduction of mTOR-S6K plays an important role in MK-2206 resistance, and its inhibitor, AZD8055, may be promising in molecular targeted therapy. Nevertheless, there is possibility that other mechanisms may contribute to MK-2206 resistance as well. In this study, GSK3β and MYC were tested to find the relationship with MK-2206 resistance, but no solid evidence was found. Meanwhile, we observed that MK-2206 did not effect in cell proliferation at 5 μM in high confluence non-resistant cells, which indicated cell to cell interaction may be anther direction, and will be introduced in our next publications.

In summary, we showed AKT played an important role in cell growth of NB cells. MK-2206 is effective in suppression of NB cell growth, but simply administration of MK-2206 might cause resistance to AKT targeted treatment. Cell growth of MK-2206-resistant sublines was related to reliance on PDK1-mTOR-S6K pathway. Both upstream (PDK1) and downstream (mTOR) AGC kinase inhibitors are inhibitive to the cell growth of MK-2206-resistant sublines. We speculate that combination of PDK1-AKT-mTOR-S6K signaling inhibitors or multi-targeted tyrosine kinase inhibitors is promising to resolve MK-2206 resistance.
