# Erratum to: PDK1-mTOR signaling pathway inhibitors reduce cell proliferation in MK2206 resistant neuroblastoma cells

**DOI:** 10.1186/s12935-015-0261-6

**Published:** 2015-11-17

**Authors:** Lei Qi, Hidemi Toyoda, Dong-qing Xu, Ye Zhou, Naoto Sakurai, Keishirou Amano, Kentaro Kihira, Hiroki Hori, Eiichi Azuma, Yoshihiro Komada

**Affiliations:** Department of Pediatrics and Developmental Science, Mie University Graduate School of Medicine, 2-174 Edobashi, Tsu, Mie 514-8507 Japan; Department of Pediatrics, Xin Hua Hospital Affiliated to Shanghai Jiao Tong University School of Medicine, 1665 Kong Jiang Road, Shanghai, 200092 China; Department of Child Health Nursing, Mie University Graduate School of Medicine, 2-174 Edobashi, Tsu, Mie 514-8507 Japan

## Erratum to: Cancer Cell Int (2015) 15:91 DOI 10.1186/s12935-015-0239-4

Unfortunately, the original version of this article [1] contained an error. After publication it came to the authors’ attention that the figure legends were displayed incorrectly. The correct figure legends can be found below in this erratum.

**Fig.** **1** MK-2206 suppressed the cell growth of NB cells. **a** MK-2206 suppressed the cell growth of NB cell lines. LAN-1, KP-N-SIFA, NB-19, and SK-N-DZ cells were cultured in RPMI1640 + 10 % FBS with MK-2206 at indicated concentrations. Cell growth was evaluated as cell numbers at 72 h, and it was repeated three times. Data are expressed as the mean (±SD). **b** Photomicrographs of MK-2206 non-resistant and resistant cells. Cells were cultured in glass bottom slide chambers with RPMI1640 + 10 % FBS, with MK-2206 (resistant sublines)/without MK-2206 (non-resistant cells) overnight. A 50 µm scale is indicated (Olympus Fluoview fv1000, DIC acquisition, ×40). **c** MK-2206 showed less inhibition in the proliferation of MK-2206-resistant sublines than in the non-resistant cells. Indicated cells were cultured in RPMI1640 + 10 % FBS with MK-2206 at indicated concentrations. Cell growth was evaluated as cell numbers at indicated hours, and it was repeated three times. Data are expressed as the mean (±SD). *P < 0.01

**Fig.** **2** MK-2206 showed less inhibition in cell growth of MK-2206-resistant sublines. **a** MK2206 suppressed cell growth in a dose dependent method, and MK-2206-resistant sublines maintained resistance after 2-week withdrawal of MK-2206. Indicated cells were cultured in RPMI1640 + 10 % FBS with MK-2206 at the indicated concentrations. Cell growth was evaluated as cell numbers at 72 h, and it was repeated three times. Data are expressed as the mean (±SD). **b** IC50 of MK-2206 in indicated cells. **c** The effect of MK-2206 on cell cycle phase distribution. LAN-1 and LAN-1-MK were treated with/without MK-2206 (5 µM) in RPMI1640 with 10 % FBS for 12 h followed by analysis of cell cycle phase distribution, as introduced in “Methods”. Cells were stained with propidium iodide (PI) for 30 min followed by FACScan flow cytometer. **d***Column chart* of cell cycle distribution in **c**

**Fig.** **3** Effect of GSK2334470 (GSK), PDK1 inhibitor, in MK-2206-resistant sublines compared with non-resistant cells. **a** Indicated cells were treated with GSK at indicated concentrations, with/without MK-2206 (5 µM) in RPMI1640 + 10 % FBS. Cell growth was evaluated as cell numbers at 72 h, and it was repeated three times. Data are expressed as the mean (±SD). **b** IC50 of GSK in indicated cells. **c** The effect of GSK on cell cycle phase distribution in LAN-1 and LAN-MK. LAN-1 and LAN-1-MK were treated with GSK (5 µM) with/without MK-2206 (5 µM) in RPMI1640 with 10 % FBS for 12 h followed by analysis of cell cycle phase distribution, as introduced in “Methods”. Indicated cells were stained with PI for 30 min followed by FACScan flow cytometer

**Fig.** **4** Effect of AZD8055 (AZD), mTOR inhibitor, in MK2206 resistant sublines compared with non-resistant cells. **a** Indicated cells were treated with AZD at indicated concentrations, with/without MK-2206 (5 µM) in RPMI1640 + 10 % FBS. Cell growth was evaluated as cell numbers at 72 h, and it was repeated three times. Data are expressed as the mean (±SD). **b** IC50 of AZD in indicated cells. **c** The effect of AZD on cell cycle phase distribution in LAN-1 and LAN-MK. LAN-1 and LAN-1-MK were treated with AZD (50 nM) with/without MK-2206 (5 µM) in RPMI1640 with 10 % FBS for 12 h followed by analysis of cell cycle phase distribution, as introduced in “Methods”. Indicated cells were stained with PI for 30 min followed by FACScan flow cytometer

**Fig.** **5** Effect of GSK2334470 (GSK) on PDK1-mTOR-S6K axis in MK-2206-resistant sublines. **a**–**d** After 1 h serum starvation, indicated cells were incubated in RPMI1640 + 10 % FBS with/without MK-2206 (5 µM) or GSK (5 µM). Phosphorylation of PDK1, AKT, mTOR, and S6K were detected by western blot at 1.5 and 12 h, so were AKT and Actin. GSK3β, p-GSK3β and N-MYC were also detected
